# Multilocus Sequence Typing and *rtx*A Toxin Gene Sequencing Analysis of *Kingella kingae* Isolates Demonstrates Genetic Diversity and International Clones

**DOI:** 10.1371/journal.pone.0038078

**Published:** 2012-05-31

**Authors:** Romain Basmaci, Pablo Yagupsky, Brice Ilharreborde, Kathleen Guyot, Nurith Porat, Marilyn Chomton, Jean-Michel Thiberge, Keyvan Mazda, Edouard Bingen, Stéphane Bonacorsi, Philippe Bidet

**Affiliations:** 1 Univ Paris Diderot, Sorbonne Paris Cité, EA 3105, Paris, France; 2 AP-HP, Laboratoire de Microbiologie, Hôpital Robert-Debré, Paris, France; 3 Clinical Microbiology Laboratory, Soroka University Medical Center, Ben-Gurion University of the Negev, Beer-Sheva, Israel; 4 Univ Paris Diderot, Sorbonne Paris Cité, Paris, France; 5 AP-HP, Service de Chirurgie Orthopédique, Hôpital Robert-Debré, Paris, France; 6 Pediatric Infectious Disease Unit, Soroka University Medical Center, Ben-Gurion University of the Negev, Beer-Sheva, Israel; 7 Plate-Forme Génotypage des Pathogènes et Santé Publique, Institut Pasteur, Paris, France; Institut de Génétique et Microbiologie, France

## Abstract

**Background:**

*Kingella kingae*, a normal component of the upper respiratory flora, is being increasingly recognized as an important invasive pathogen in young children. Genetic diversity of this species has not been studied.

**Methods:**

We analyzed 103 strains from different countries and clinical origins by a new multilocus sequence-typing (MLST) schema. Putative virulence gene *rtx*A, encoding an RTX toxin, was also sequenced, and experimental virulence of representative strains was assessed in a juvenile-rat model.

**Results:**

Thirty-six sequence-types (ST) and nine ST-complexes (STc) were detected. The main STc 6, 14 and 23 comprised 23, 17 and 20 strains respectively, and were internationally distributed. *rtx*A sequencing results were mostly congruent with MLST, and showed horizontal transfer events. Of interest, all members of the distantly related ST-6 (n = 22) and ST-5 (n = 4) harboured a 33 bp duplication or triplication in their *rtx*A sequence, suggesting that this genetic trait arose through selective advantage. The animal model revealed significant differences in virulence among strains of the species.

**Conclusion:**

MLST analysis reveals international spread of ST-complexes and will help to decipher acquisition and evolution of virulence traits and diversity of pathogenicity among *K. kingae* strains, for which an experimental animal model is now available.

## Introduction

Increasing use of improved culture methods and sensitive nucleic acid amplification assays in recent years has resulted in the recognition of *Kingella kingae* as a common etiology of skeletal system infections and bacteremia below the age of 3 years [1]–[11]. The organism is carried asymptomatically in the oropharyngeal mucosal surfaces from which it may be transmitted from-person-to-person and/or penetrate into the bloodstream and disseminate to remote sites such as bones, joints or the endocardium [12]–[16] and, more rarely, to the meninges or the peritoneal cavity [17]–[19].

Because the organism was traditionally considered an exceptional cause of human disease, our current knowledge on the population structure of the species and potential differences in terms of virulence between strains is still limited. A study by Lehours demonstrated polymorphism of the *rtx*A gene, which encodes the RTX toxin [10]. Genotyping of 240 strains derived from healthy carriers by pulsed-field gel electrophoresis (PFGE) demonstrated 40 distinct clones of which a few were undistinguishable from those detected among isolates from patients with invasive infections [20]. Although these preliminary results indicate that *K. kingae* exhibits genetic heterogeneity, development of a highly reproducible genotyping method is necessary to decipher the genetic organization of the species and determine whether certain genotypes are associated with either carriage or disease. Furthermore, to date the differences in pathogenicity of *K. kingae* strains have only been studied *in vitro* and no animal model of invasive infection has been developed. Our animal model may contribute to discriminate between highly and low virulent clones and would point to clones to be further investigated to identify genetic determinants of virulence.

In the present study we studied the genetic diversity of 103 *K. kingae* isolates from different countries and different clinical sources by use of a new multi-locus sequence-typing (MLST) scheme. We also analysed the relationship between MLST clustering and the diversity of the *rtx*A encoding gene. Finally we developed an animal model to assess the virulence of *K. kingae* isolates.

## Materials and Methods

### Bacterial strains

To develop an adequate MLST scheme, a diverse collection of *K. kingae* organisms was assembled to include strains derived from individuals living in different geographic locations and isolated in different periods, patients with a variety of clinical syndromes, as well as epidemiologically-unrelated healthy carriers.

Seven *K. kingae* strains, available in international reference collections (4 from France and 3 from Norway), were obtained from the Pasteur Institute of Paris (Table S1). In addition, 25 clinical isolates from 3 different French hospitals were also studied. These isolates derived from 23 patients with osteoarticular infections (OAI), bacteremia (n = 1) and endocarditis (n = 1) (Table S2).

Over the years, the Clinical Microbiology Laboratory of the Soroka University Medical Center (SUMC) of southern Israel has gathered over 1,000 *K. kingae* isolates from respiratory carriers living in the region, as well as 170 organisms from Israeli patients with a variety of invasive *K. kingae* infections. To assure a wide selection of Israeli strains, 69 candidates for MLST typing were chosen on the basis of their dissimilar PFGE profiles and clinical and epidemiological data partially published elsewhere [20]. Whenever it was possible, pairs of invasive and colonizing isolates exhibiting indistinguishable PFGE profiles were selected. Overall, 69 Israeli isolates were studied, including 33 derived from asymptomatic carriers, 17 from patients with OAI, bacteremia (n = 10), and endocarditis (n = 9) (Table S3).

Two isolates from the USA (kindly provided by Ruth Lynfield and Joseph W. St. Geme), and one isolate from a healthy Russian pediatric carrier, were also studied. (Table S3).

Organisms were cultured on chocolate Isovitalex agar or Columbia blood agar for 24 to 48 h at 37°C in a 5% CO_2_-enriched athmosphere.

### DNA extraction, amplification and sequencing

DNA was extracted from specimens with the BioRobot EZ1 workstation using the EZ1 DNA tissue kit (Qiagen, Courtaboeuf, France) according to the manufacturer's recommendations, and stored at −80°C.

For DNA amplification, the PCR mixture contained 25 µL of AmpliTaq Gold PCR MasterMix (Life Technologies, Villebon sur Yvette, France), 2 µL (10 µM) of each primer (forward and reverse), 19 µL of pure water and 2 µL of DNA (∼5 ng) in a final volume of 50 µL. Amplification was performed on an iCycler (Bio-Rad, Marnes la Coquette, France), with an initial step of 15 min at 95°C, followed by 35 cycles of 15 s at 95°C, 30 s at 56°C, 1 min 30 s at 72°C, and a final extension step of 10 min at 72°C. Amplification products were stored at 4°C until further tested. Amplification products were visualized under U.V. after migration on gel electrophoresis, and both strands were sequenced by a Beckman-Coulter Genomics instrument (Takeley, Essex, United Kingdom).

### MLST scheme

At the beginning of the study no *Kingella* species had been sequenced with the exception of one *K. oralis* isolate. We first selected 7 housekeeping genes (*abc*Z, *adk*, *aro*E, *cpn*60, *gdh*, *rec*A and *fum*C) that were known for *K. kingae* (*cpn*60) [7], or used for related species MLST such as *N. meningitidis* (*abc*Z, *adk*, *aro*E, *fum*C and *gdh*) or *H. influenzae* (*rec*A) and that were common to *K. oralis*. Consensus primers were designed by alignment of *N. meningitidis* and *K. oralis* sequences, available on database. Then they were tested on 4 *K. kingae* strains (ATCC 23330, ATCC 23331, CIP 73.1 and CIP 102473) and amplification products were sequenced, allowing us to design new consensus primers for each *K. kingae* gene.

In addition to the MLST scheme, the *rtx*A gene was also amplified using primers described by Lehours *et* al. and sequenced [10].

### Estimation of the between-strains relatedness

A different allele number was given to each distinct sequence within a locus, and a distinct sequence-type (ST) number was attributed to each distinct alleles combination. Isolates were grouped into ST-complexes (STc) if they differed at no more than one locus from at least one other member of the group. Founder genotypes of STcs were defined as the ST of the STc with the highest number of neighboring STs (single locus variants). Molecular epidemiological data were stored and analysed using software BioNumerics (Version 6.6; Applied-Maths, Belgium). Relatedness between the different STs was investigated based on comparison of allelic profiles using the minimum spanning tree (MStree) method from BioNumerics. The relatedness between each *rtx*A sequence was shown as a dendrogram, constructed by the UPGMA method using MEGA 3.1 program [21].

### Animal model

After 24 h of culture on chocolate Isovitalex agar strains were suspended in phosphate-buffered saline (PBS) at an optic density at 600 nm of 1.2, corresponding to a concentration of approximately 10^8^ CFU/mL. Determination of bacterial concentration was retrospectively performed by serial dilution and plating.

One hundred µL of these suspensions (10^7^ CFU) were injected intraperitoneally in the left inferior quadrant of 5-day-juvenile albino Spragues Dawley rats (Charles River Laboratories, France). Inoculated animals were examined daily for 5 consecutive days for skin discoloration, signs of peritonitis and survival rates. If subcutaneous lesions were observed, a piece of tissue was removed, minced, suspended in 500 µL of brain-heart infusion and plated onto chocolate agar.

### Ethics statement

All experiments and animals housing procedures complied with the University Paris Diderot animal ethics committee guidelines and were approved by local animal ethics committee “Comité d'Éthique Expérimentation Animale Bichat-Debré” (2011-14/676-0054).

### Statistical Analysis

Disease-free survival curves were compared by Log-rank Test. P-value below 0.05 was considered to denote significant difference.

## Results

### Housekeeping genes characteristics (Table 1)

**Table 1:** Characteristics of genes studied and primers used in this study.

Gene^a^	Primers forward (F) and reverse (R)	Products	Analyzed	Number
		(bp)	sequence	of alleles
			(bp)	
*abc*Z	(F)TGACGACCAAGCGAGCGTGTTTGA	531	453	16
	(R)TCCGCCTCAACCGCCAATTCCT			
*adk*	(F)CACAAGCGCAATTTATTACGCGCGA	434	351	11
	(R)GTCGTCATCGCGTTGCACCAAGT			
*aro*E	(F)TTTGCCGCACAAGAAGGTGCGCAA	587	519	15
	(R)GGCTGGTTCGCATAAAACATATCG			
*cpn*60	(F)TGTTGGCGCAAGCGATTGTTGCT	379	303	8
	(F)^b^AAACCAATGATGTGGCTGGCGACG			
	(R)AATGGGCTGTCCAAACCAGCGAT			
*gdh*	(F)CGGCGCGTTGCGCGATATGGTGCA	657	576	13
	(R)CCAGTTGTCCAAAATCGGCATCAC			
*rec*A	(F)GACGAAGAATTGCAAGTCATTTCCA	433	354	6
	(R)AGTTTACGCAAGGCTTGGCTCATC			
*rtx*A	(F)GCCGAATGGGAAGATTTCTG	1198	909	22
	(R)GCATTCATAAACGCCAACG			

a : Gene function, *abc*Z (ABC transporter), *adk* (Adenosine kinase), *aro*E (Shikimate dehydrogenase), *cpn*60 (Heat shock protein), *gdh* (Glucose-6-phosphate dehydrogenase), *rec*A (Recombination protein A), *rtx*A (Haemolysin).

b : Alternative *cpn*60-forward-primer in case of failing amplification.

Among the 7 genes initially selected, *fum*C was abandoned because we did not succeed to amplify it in *K. kingae*. Therefore, 6 couples of primers were designed after analyzing the sequence of the target genes (*abc*Z, *adk*, *aro*E, *cpn*60, *gdh* and *rec*A) and allowed amplification of all but 6 strains. For 5 of them, an alternative *cpn*60-forward-primer was designed and allowed sequencing. Only one strain (PV1572 from a healthy carrier) was unsuccessfully sequence-typed by failing of *aro*E amplification. Lengths of analyzed sequences were between 303 bp (*cpn*60) and 576 bp (*gdh*). The number of alleles is indicated in Table 1 and ranged from 6 (*rec*A) to 16 (*abc*Z). Allele sequences of each gene for all strains are available on the Pasteur Institute of Paris website http://www.pasteur.fr/recherche/genopole/PF8/mlst/Kingella_kingae.html.

### Estimating relatedness between the strains

**Figure 1:**
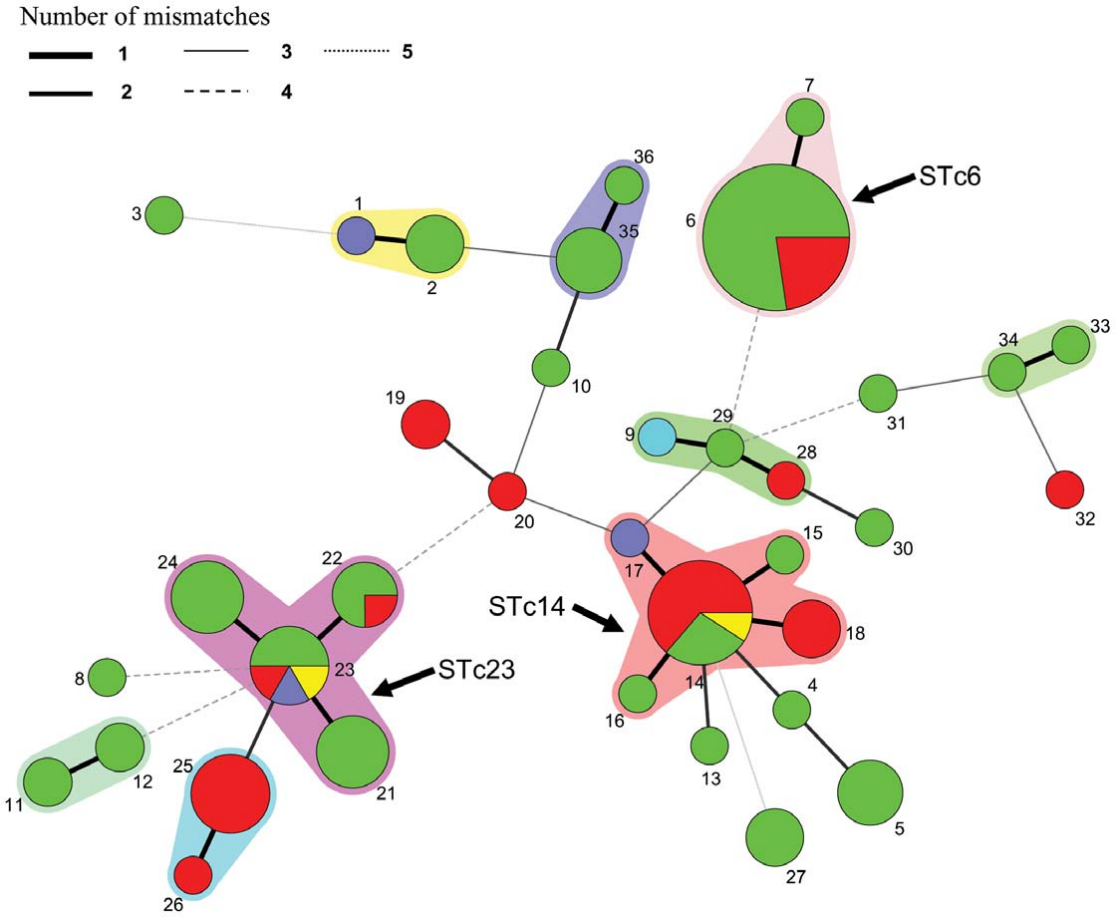
Minimum spanning tree analysis, using BioNumerics Version 6.6, of the 103 *K. kingae* isolates based on allelic profiles of 6 housekeeping genes. Each circle corresponds to a sequence type (ST). The ST number is given beside the circle, and the size of the circle is related to the number of isolates found with that profile (from 1 for small circles e.g. ST-1 to 22 for ST-6). Each colour inside circles represents geographical origin of the strains (green: Israel; red: France; blue: Norway; yellow: USA and turquoise: Russia). Colored zones between some groups of circles indicate that these profiles belong to the same ST complex. The three major ST complex are indicated. Width of line joining two STs indicates the number of alleles differing.

The analysis of the sequences of the 6 housekeeping genes for the 103 *K. kingae* isolates revealed 36 STs, among them 21 were unique (Table 2). The 3 most common STcs (STc-6, STc-14 and STc-23) included 60/103 (58.3%) of all typed isolates. These three STcs were distantly related (Figure 1), as STc-14 shared no common allele with the two others and STc-6 shared only one common allele with STc-23. STc-6 was mainly composed of one ST (ST6, n = 22; ST7, n = 1) whereas STc-23 (n = 20) and STc-14 (n = 17) were more diverse and included STs-21-22-23-24, and STs-14-15-16-17-18, respectively. Of note, the type strain (ATCC 23330) was the only strain of ST-1 which belong to STc-1 (n = 4) and was distantly related to the most frequent STcs (Figure 1).

**Table 2:** Correspondences between MLST, *rtx*A alleles and geographic origins of 103 *K. kingae* strains.

ST^a^	ST complex	*rtx*A	Number of	Countries of isolation^b^ (n)
		alleles	strains	
1	1	16	1	Norway (ATCC 23330)
2	1	1	3	Is (3)
3		14	1	Is (1)
4		14	1	Is (1)
5		17	4	Is (4)
6	6	8; 9; 10; 11	22	Is (17), Fr (5)
7	6	15	1	Is (1)
8		1	1	Is (1)
9	29	4	1	Russia (1)
10		6	1	Is (1)
11	11	18	2	Is (2)
12	11	18	2	Is (2)
13		14	1	Is (1)
14	14	14	11	Fr (7, including CIP 73.01 and CIP 101722)
				Is (3), USA (1)
15	14	14	1	Is (1)
16	14	14	1	Is (1)
17	14	4	1	Norway (ATCC 23332)
18	14	14	3	Fr (3)
19		7	2	Fr (CIP 102470; CIP 102473)
20		7	1	Fr (1)
21	23	1	5	Is (5)
22	23	1	4	Is (3), Fr (1)
23	23	1; 2; 12	6	Is (3), Fr (1), Norway (ATCC 23331), USA (1)
24	23	12	5	Is (5)
25	25	1	6	Fr (6)
26	25	1	1	Fr (1)
27		5	3	Is (3)
28	29	4	1	Fr (1)
29	29	4	1	Is (1)
30		4	1	Is (1)
31		3	1	Is (1)
32		3	1	Fr (1)
33	33	3	1	Is (1)
34	33	13	1	Is (1)
35	35	6	4	Is (4)
36	35	6	1	Is (1)

a : ST, sequence-type;

b : Is, Israel; Fr, France.

Some STcs were worldwide distributed. STc-6 included 18 isolates from Israel and 5 from France, and STc-14 and STc-23 included isolates from Israel, France, Norway, and the USA, whilst some STcs seemed to be country-specific, such as the exclusive French STc-25, representing 7/29 (24.1%) of the French strains (Table 2 and Figure 1).

### Polymorphism of *K. kingae* rtxA gene

**Figure 2:**
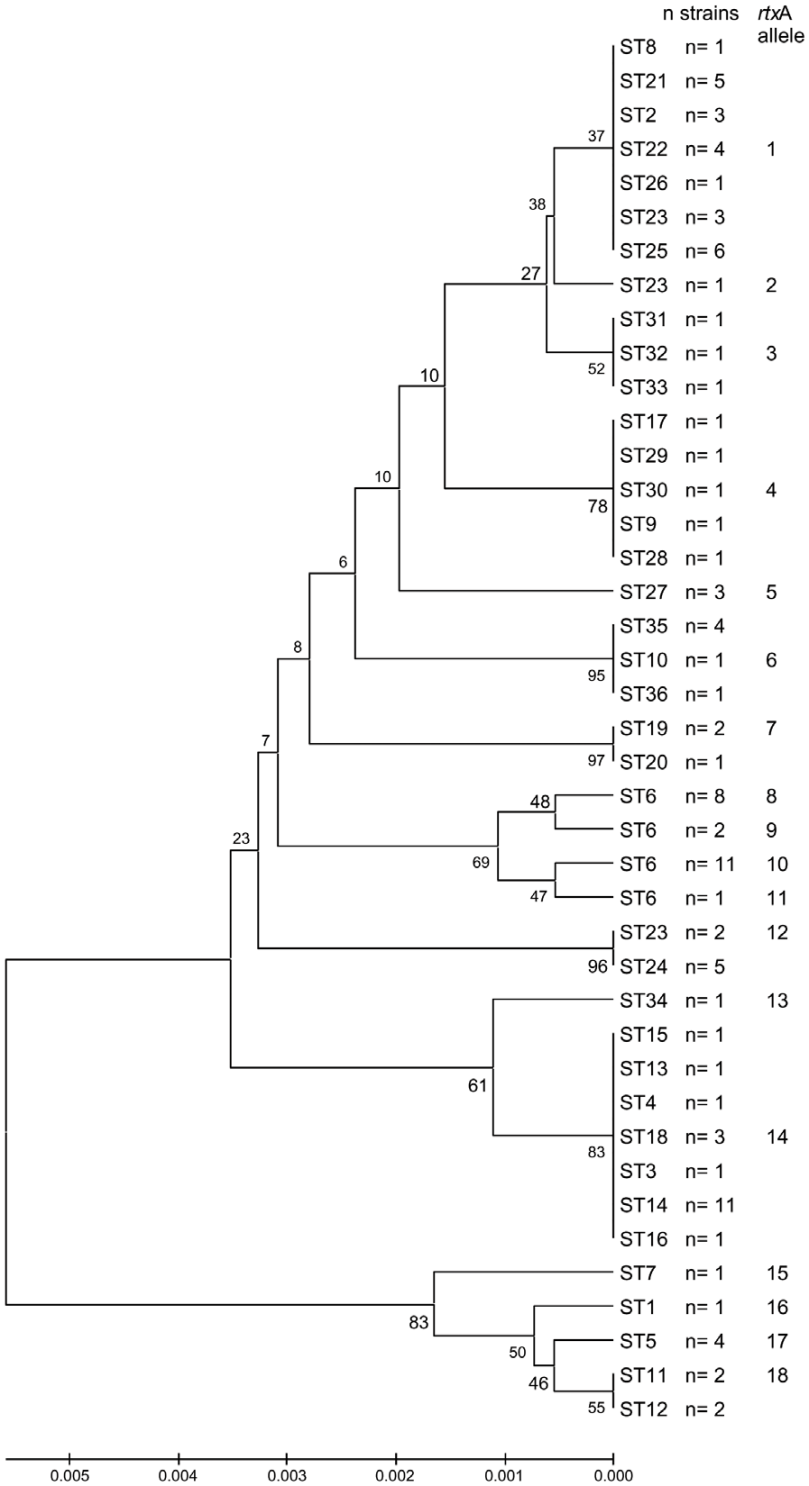
Dendrogram of *rtx*A sequences using UPGMA method. Distribution of 103 strains according to their ST among the 18 alleles is also indicated. All the strains with alleles 8, 10, 11 and 17 have a duplication of 33 bp, and all allele 9 strains have a triplication (see Figure 3).

To examine the potential relationships between sequence polymorphism of the core genome and that of a putative virulence gene, we studied sequences of the haemolysin gene *rtx*A and compared it with the MLST results. A sequence ranging from 909 to 975 bp was analyzed for the gene *rtx*A for the 103 *K. kingae* strains.. The mean G+C% of this gene was strikingly different from that of the MLST schema (37.6% vs. 49.7% respectively) and from that of the largest contig (47,149 bp) of the recently sequenced *K. kingae* type strain (accession number AFHS01000014.1, G+C% = 48.1%). The genetic relatedness of *rtx*A sequences is shown as a dendrogram (Figure 2) and in Table 2. This gene was remarkably polymorphic and exhibited 18 different sequences. The 18 sequences are available on NCBI database under accession number JQ340459 to JQ340476. Four *rtx*A alleles (15 to 18) found in 10 strains, constituted a subgroup distantly related to the 14 others (Figure 2). This may suggest that the 10 strains had acquired their *rtx*A genes from a source different from that of other *K. kingae* strains. However none of the *rtxA* alleles had a significant homology to sequences in GenBank database. *rtx*A sequencing results were congruent in most strains with their allocation to ST and STc by MLST. For instance, *rtx*A-allelles 8 to 11 included all and exclusively strains of the major ST-6 (Figure 2). However, incongruence was observed for several alleles: the *rtx*A-allele 14 was dispersed in unrelated ST-3 and STc-14, and the *rtx*A-allele 1 was distributed among the distantly related ST-2, ST-8, STc-23 and STc-25; the strain belonging to ST-17 was the only member of STc-14 that had the same *rtx*A allele found in STc-29. Finally, the 4 *rtx*A alleles (15 to 18) constituting a subgroup, were scattered among unrelated STs. Altogether, these results suggest that horizontal transfers of *rtx*A alleles had occurred in *K. kingae* species however the random occurrence of multiple advantageous mutations in the rtxA genes in strains belonging to distantly related STs can not be excluded.

**Figure 3:**
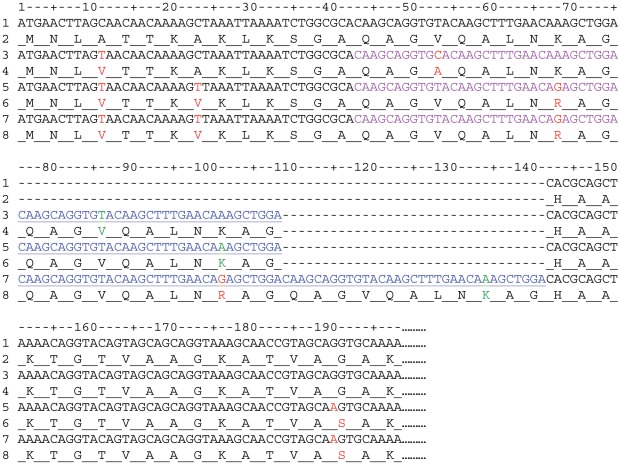
Alignment of the 5′ extremity of *rtx*A gene and corresponding amino acids of 4 *Kingella kingae* strains. For each strain, first line represents nucleotide-sequence; second line represents aminoacid sequence. Lines 1 and 2: type strain ATCC 23330; lines 3 and 4: strain BB631 of ST-5 with 33 bp duplication; lines 5 and 6: strain SIL of ST-6 with 33 bp duplication; lines 7 and 8: HAM of ST-6 with 33 bp triplication. Mismatches compared to type strain are indicated in red; the inserted sequences are indicated in blue and underlined; the original sequence, which is duplicated, is in purple. Mismatches between duplicated-inserted sequences and the corresponding original sequences are indicated in green.

Of note, in 26 *rtx*A sequences, we observed an insertion of 33 bp, corresponding to 11 amino-acids, which was repeated twice in two isolates (HAM and KK6) (Figure 2 and 3). Interestingly, all these strains belonged to two unrelated STs, ST-6 and ST-5 (Figure 1). The location and the sequence of this insertion were identical for all strains, except HAM and KK6, and appeared in nucleotide 76 (Figure 3). This insertion is a quasi perfect duplication of the immediately previous 33 bp, corresponding to following aminoacid sequence QAG(V/A)QALN(R/K)AG (Figure 3). Moreover, *rtx*A sequences were very different between strains of ST-5 and ST-6 (Figure 2). These observations are not in favour of a horizontal transfer of *rtx*A genes between these two STs, which could have explain their similar particular genetic traits, but plead for the occurrence of two unrelated insertion events.

### Animal model

**Figure 4:**
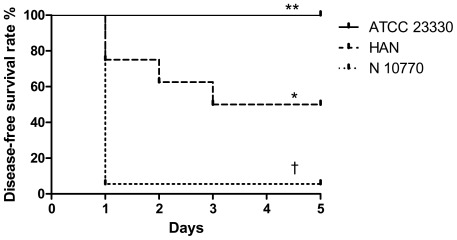
Disease-free survival curve of three groups of 16 juvenile-rats after intraperitoneal inoculation with 3 different isolates of *Kingella kingae* (ATCC 23330: carriage, HAN: osteoarthritis and N10770: bacteremia). *: *P = 0.001 vs.* ATCC 23330 by Log-rank Test; **: *P<0.001 vs.* N10770 by Log-rank Test; †: *P<0.001 vs.* HAN by Log-rank Test.

To determine whether differences of virulence exist between *K. kingae* isolates, we infected three groups of eight 5-day-juvenile-rats with 3 MLST-unrelated-isolates of different clinical origin. We selected the strains ATCC 23330 (type strain, isolated from the nose, unique strain of the ST-1), HAN (isolated from a patient with OAI and belonging to the major ST-6) and N10770 (from a patient with bacteremia, belonging to ST-28, and representing a minor invasive ST). The inocula injected intraperitoneally were 10^7^ CFU for each strain. The experiment was repeated twice and results are presented pooled. The disease-free survival curve showed significant differences (Figure 4). Animals infected with strain ATCC 23330 did not present any sign of infection at any time and were all alive and healthy at day 5. In contrast, in the N10770 inoculted rats, 10 animals were dead at 24 h, and five of the six surviving animals had a major ischemic-necrosis lesion in the abdomen that engulfed their left hind leg. These five animals were then sacrificed. The remaining rat was alive and healthy at day 5. The HAN strain displayed an intermediate virulence, all the 16 animals were alive at 48 h, but 6 had clinical symptoms of infection: five had an abscess on the abdomen and one looked ill. Aspiration of the abscesses revealed polymorphonuclear leucocytes and viable *K. kingae* organisms. Four animals died between days 3 and 5, and 8 animals remained healthy.

## Discussion

To date, the population structure of the emerging pathogen *K. kingae* remains largely unknown. In this study we established, for the first time, a MLST schema of the species and observed a wide genetic diversity with 36 distinct STs and 9 STcs among 103 isolates. We demonstrated that 3 distinct STcs (STc-6, STc-14 and STc-23), sharing almost no common alleles and including 58.3% of all isolates, were represented in geographically distant countries, suggesting that these strains probably posses a selective biologic advantage and have evolved independently.

Whilst the difference between the G+C% of core genome and *rtx*A suggest that the haemolysin-encoding gene had been acquired from a different bacterial species, important congruencies observed suggest a remote acquisition and co-evolution of the *rtx*A gene and the genes examined for MLST typing. We also noted a duplication of 33 bp in some sequence types of the *rtx*A gene, which were previously described by Lehours *et* al. [10]. Interestingly, duplications occurred exclusively among ST-6 and ST-5 strains. However, we made a different interpretation of the genetic event to that Lehours *et* al. Indeed, we found that this insertion occurred in position 76 and not in position 67, and, although a single non-synonymous mutation was detected, we concluded that this insertion is a duplication of the immediately adjacent 33 bp fragment. Furthermore, considering that sequences out of the insertion site were different between both STs, we hypothesized that this genetic trait arose through convergent evolution, and probably not by horizontal transfer of the *rtx*A gene, confering a selective biological advantage. Whether the genetic structure of this 33 bp sequence promotes duplication independently of any selective advantage remains to be determined.

To our knowledge, we developed the first experimental model of *K. kingae* infections. This model of intraperitoneal infection in juvenile rats demonstrated clear differences in virulence between wild strains and the ATCC 23330 type strain, which exhibited the lowest virulence. Whereas this organism isolated in the 1960's from the respiratory tract was originally less invasive or rather experienced genome reduction because of repeat subcultures over the years is unknown. The two other strains examined exhibited intermediate and high degree of virulence. Therefore, the herein described animal model can serve to identify virulent clones among candidates from epidemiological data in order to determine novel potential virulence factors.

In conclusion, our MLST tool will serve future epidemiological studies and transcontinental comparisons providing a comprehensive view of circulating clones and detecting emerging ones. Moreover, the MLST data may be used for targeting clones to be further investigated for virulence in our animal model. We also suggest that the recent fully sequenced ATCC 23330 type strain, which appears devoid of pathogenicity in our animal model, may serve for future comparative genomic studies.

## Supporting Information

Table S1 **Strains of** ***Kingella kingae*** **available on international collections used in the study.**
(DOC)

Table S2 **Twenty-five French clinical** ***Kingella kingae*** **isolates.**
(DOC)

Table S3 **Seventy-one** ***Kingella kingae*** **isolates, typed by MLST from Israel, Russia and USA.**
(DOC)
