# Supplementary material for: Multilocus Sequence Typing and rtxA Toxin Gene Sequencing Analysis of Kingella kingae Isolates Demonstrates Genetic Diversity and International Clones
Source: PLoS One. 2012 May 31;7(5):e38078. doi: 10.1371/journal.pone.0038078 (PMC3365011; doi:10.1371/journal.pone.0038078)
Supplement: Table S1 — Strains of Kingella kingae available on international collections used in the study. (DOC) [file pone.0038078.s001.doc]

Table S1: Strains of *Kingella kingae* available on international collections used in the study.

| Reference number of the strain | Clinical condition | Geographical isolation | Year of isolation |
| --- | --- | --- | --- |
| ATCC 23330 (CIP 80.16) | Carriage | Oslo, Norway | 1966 |
| ATCC 23331 (CIP 68.12) | Bacteremia | Oslo, Norway | 1968 |
| CIP 73.01 | Bacteremia | Besançon, France | 1972 |
| CIP 102473 | OAI^a^ | Paris, France | 1986 |
| CIP 101722 | Bacteremia | Grenoble, France | 1985 |
| CIP 102470 | OAI | Paris, France | 1986 |
| ATCC 23332 (CIP 80.18) | Bacteremia | Oslo, Norway | 1968 |

a: Osteoarticular infection
