# Supplementary material for: Multilocus Sequence Typing and rtxA Toxin Gene Sequencing Analysis of Kingella kingae Isolates Demonstrates Genetic Diversity and International Clones
Source: PLoS One. 2012 May 31;7(5):e38078. doi: 10.1371/journal.pone.0038078 (PMC3365011; doi:10.1371/journal.pone.0038078)
Supplement: Table S2 — Twenty-five French clinical Kingella kingae isolates. (DOC) [file pone.0038078.s002.doc]

Table S2: Twenty-five French clinical *Kingella kingae* isolates

| Isolate number | Abbreviation used | Clinical diagnosis | Year | Geographical Isolation (Hospital, City) |
| --- | --- | --- | --- | --- |
| WAR 26245 | WAR | OAI^a^ | 2007 | Robert-Debré, Paris |
| OUA 30390 | OUA | OAI | 2010 | Robert-Debré, Paris |
| MEN 26437 | MEN | OAI | 2007 | Robert-Debré, Paris |
| HAN 26314 | HAN | OAI | 2007 | Robert-Debré, Paris |
| BOU 30672 | BOU | OAI | 2010 | Robert-Debré, Paris |
| HER 31223 | HER | OAI | 2010 | Robert-Debré, Paris |
| SCH 25972 | SCH | OAI | 2007 | Robert-Debré, Paris |
| BIA 29991 | BIA | OAI | 2009 | Robert-Debré, Paris |
| BUS 25298 | BUS | OAI | 2007 | Robert-Debré, Paris |
| MAT 26683 | MAT | OAI | 2008 | Robert-Debré, Paris |
| N10-6602 | N6602 | OAI | 2010 | Nantes, Nantes |
| N10-10419 | N10419 | OAI | 2010 | Nantes; Nantes |
| N10-9223 | N9223 | OAI | 2010 | Nantes, Nantes |
| N10-10770 | N10770 | Bacteremia | 2010 | Nantes, Nantes |
| N10-6318 | N6318 | OAI | 2010 | Nantes, Nantes |
| S1003337 | S37 | OAI | 2010 | Trousseau, Paris |
| S1100633 | S33 | OAI | 2011 | Trousseau, Paris |
| R4599 | R99 | OAI | 2011 | Trousseau, Paris |
| MAR 1853 | MAR | OAI | 2011 | Robert-Debré, Paris |
| SIL 32489 | SIL | OAI | 2011 | Poissy, Poissy |
| TEI 137111 | TEI | OAI | 2011 | Robert-Debré, Paris |
| HAM 137138 | HAM | OAI | 2011 | Robert-Debré, Paris |
| POH 14284 | POH | OAI | 2011 | Robert-Debré, Paris |
| WAN 11661 | WAN | OAI | 2011 | Robert-Debré, Paris |
| SAI 11985 | SAI | OAI | 2011 | Robert-Debré, Paris |

a: OAI, osteoarticular infection
