# Supplementary material for: Multilocus Sequence Typing and rtxA Toxin Gene Sequencing Analysis of Kingella kingae Isolates Demonstrates Genetic Diversity and International Clones
Source: PLoS One. 2012 May 31;7(5):e38078. doi: 10.1371/journal.pone.0038078 (PMC3365011; doi:10.1371/journal.pone.0038078)
Supplement: Table S3 — Seventy-one Kingella kingae isolates, typed by MLST from Israel, Russia and USA. (DOC) [file pone.0038078.s003.doc]

Table S3: Seventy-one *Kingella kingae* isolates, typed by MLST from Israel, Russia and USA

| Patient status and characteristics of isolates | | | | |
| --- | --- | --- | --- | --- |
| Healthy Carriers | |  | Invasive Diseases |  |
| Isolate number | Geographical isolation | Isolate number | Clinical diagnosis | Geographical isolation |
|  |  |  |  |  |
| BB114 | Israel (south) 2006 | KK245 | Bacteremia | Israel (south) 2005 |
| KK86 | Israel (south) 1994 | K81 | OAI^a^ | Israel (south) 1991 |
| KK203 | Israel (south) 2004 | KK256 | Tenosynovitis/Bacteremia | Israel (south) 2007 |
| BB728 | Israel (south) 2006 | KK409 | Endocarditis | Israel (east) 2010 |
| KK113 | Israel (south) 1996 |  |  |  |
| BB060 | Israel (south) 2006 | KK60 | Endocarditis | Israel (south) 1994 |
|  |  | KK88 | OAI | Israel (north) 1996 |
| AA207 | Israel (south) 2006 | KK131 | Bacteremia | Israel (south) 1997 |
| AA046 | Israel (south) 2006 |  |  |  |
| CC388 | Israel (north) 2006 |  |  |  |
| KK3 | Israel (south) 1994 |  |  |  |
| BB012 | Israel (south) 2006 |  |  |  |
| Vir5453 | Israel (north) 2006 |  |  |  |
| KK12 | Israel (south) 1994 | KK64 | OAI | Israel (north) 199? |
|  |  | KK70 | OAI | Israel (south) 1993 |
|  |  | KK83 | OAI | Israel (south) 1991 |
|  |  | KK127 | OAI | Israel (north) 199? |
|  |  | KK412 | OAI | USA St. Louis 2004 |
| CC013 | Israel (south) 2006 |  |  |  |
| AA105 | Israel (south) 2006 |  |  |  |
| AA255 | Israel (north) 2006 |  |  |  |
| KK6 | Israel (south) 1994 | KK71 | Bacteremia | Israel (south) 1994 |
| KK107 | Israel (south) 1996 | KK101 | OAI | Israel (south) 1992 |
| AA417 | Israel (south) 2006 | KK145 | OAI | Israel (east) 1998 |
|  |  | KK156 | OAI | Israel (south) 2002 |
|  |  | KK199 | Endocarditis | Israel (north) 2004 |
|  |  | KK260 | LTB^b^/Bacteremia | Israel (south) 2007 |
|  |  | KK274 | OAI | Israel (south) 2009 |
|  |  | KK411 | Endocarditis | Israel (east) 2008 |
| D7517 | Israel (south) 2007 |  |  |  |
| AA068 | Israel (south) 2007 | KK75 | OAI | Israel (south) 1991 |
|  |  | KK141 | OAI | Israel (south) 1998 |
|  |  | KK158 | OAI/bacteremia | Israel (south) 1999 |
|  |  | KK180 | Endocarditis | Israel (center) 2002 |
|  |  | KK189 | Bacteremia | Israel (south) 2002 |
| BB016 | Israel (south) 2006 | KK97 | Bacteremia | Israel (south) 1992 |
| BB463 | Israel (south) 2007 |  |  |  |
| D2363 | Israel (south) 2007 |  |  |  |
| PV1748 | Israel (south) 2007 | KK56 | OAI | Israel (south) 1994 |
| PV1746 | Israel (south) 2006 | KK174 | Bacteremia | Israel (south) 1998 |
| CC254 | Israel (south) 2006 | KK242 | Bacteremia | Israel (south) 2005 |
| AA024 | Israel (south) 2007 |  |  |  |
| BB307 | Israel (south) 2006 |  |  |  |
| BB631 | Israel (south) 2006 | KK171 | OAI | Israel (north) 2001 |
| KK224 | Israel (center) 2005 | KK183 | OAI | Israel (north) 2002 |
| KK225 | Israel (center) 2005 |  |  |  |
|  |  | KK223 | OAI | Israel (center) 2005 |
|  |  | KK93 | Bacteremia | Israel (south) 1995 |
|  |  | KK128 | Endocarditis | Israel (south) 199? |
|  |  | KK190 | Endocarditis | Israel (south) 2002 |
|  |  | KK197 | Endocarditis | Israel (center) 2003 |
|  |  | KK238 | OAI | USA Minnesota 2003 |
|  |  | KK240 | OAI | Israel (south) 2005 |
| KK194 | Russia/Leningrad (2003) |  |  |  |

a: OAI, osteoarticular infection; b: LTB, laryngo-tracheo-bronchitis
